# The TSPO-specific Ligand PK11195 Protects Against LPS-Induced Cognitive Dysfunction by Inhibiting Cellular Autophagy

**DOI:** 10.3389/fphar.2020.615543

**Published:** 2021-01-29

**Authors:** Nannan Lan, Yongxin Liu, Zhaodong Juan, Rui Zhang, Baoyu Ma, Keliang Xie, Lina Sun, Hao Feng, Meng Sun, Jianfeng Liu

**Affiliations:** Shandong Provincial Medicine and Health Key Laboratory of Clinical Anesthesia, School of Anesthesiology, Weifang Medical University, Weifang, China

**Keywords:** lipopolysaccharide, autophagy, PK11195, perioperative neurocognitive disorders, translocator protein

## Abstract

Perioperative neurocognitive disorders (PND) is a common postoperative neurological complication. Neuroinflammation is a major cause that leads to PND. Autophagy, an intracellular process of lysosomal degradation, plays an important role in the development and maintenance of nervous system. PK11195 is a classic translocator protein (TSPO) ligand, which can improve the cognitive function of rats. In this study, we evaluate the protective effect of PK11195 on the learning and memory of rats. A rat model of lipopolysaccharide (LPS)-induced cognitive dysfunction was established by intraperitoneal injection of LPS. Morris Water Maze (MWM), Western blot, qRT-PCR, confocal microscopy and transmission electron microscopy (TEM) were used to study the role of TSPO-specific ligand PK11195 in LPS-activated mitochondrial autophagy in rat hippocampus. We found that PK11195 ameliorated LPS-induced learning and memory impairment, as indicated by decreased escape latencies, swimming distances and increased target quadrant platform crossing times and swimming times during MWM tests. TSPO, ATG7, ATG5, LC3B and p62 protein and mRNA expression increased in the hippocampus of PND model rats. The hippocampal microglia of PND model rats also have severe mitochondrial damage, and a large number of autophagosomes and phagocytic vesicles can be seen. PK11195 pretreatment significantly decreased the expression of TSPO, ATG7, ATG5, LC3B and p62 protein and mRNA, as well as mitochondrial damage. These findings suggested that PK11195 may alleviate the damage of LPS-induced cognitive dysfunction of rats by inhibiting microglia activation and autophagy.

## Introduction

Perioperative neurocognitive disorders (PND) is a common postoperative neurological complication that affects all aspects of cognitive function (He et al., 2019) such as learning, memory, attention and executive function. PND includes acute postoperative mental disorder and long-term persistent postoperative cognitive dysfunction (Evered et al., 2018). Neuroinflammation is a major cause that leads to PND. Lipopolysaccharide (LPS), the main component of the cell wall of gram-negative bacteria (Takeuchi and Akira, 2010), can produce neuroinflammation and promote cell apoptosis to cause cognitive impairment (Bossu et al., 2012; Zhang et al., 2015). Intraperitoneal injection of LPS can effectively establish an animal model of PND (Anaeigoudari et al., 2015; Satomoto et al., 2018; Skvarc et al., 2018). At present, researches on cognitive impairment are mainly focused on neuroinflammatory response (Yuan et al., 2019), structural and functional integrity disorders of blood-brain barrier (Mansour et al., 2019), Aβ metabolic disorders (Wang et al., 2020) and cholinergic dysfunction (Shin et al., 2019), but its mechanism is still unclear. The data on the link between autophagy and PND is scarce. Li et al. found that 1.5% Isoflurane exposure may impair the spatial cognitive function and the formation of hippocampal phagophore in rats (Li et al., 2015). Zhang et al. also found that autophagy is involved in cognitive dysfunction in rats induced by sevoflurane anesthesia (Zhang et al., 2016).

Autophagy is a cell clearance process in eukaryotic cells, which plays a role in maintaining cell homeostasis by dynamic degradation of damaged organelles and aberrant proteins (Glick et al., 2010). The mechanism of autophagy is generally described as formation of autophagosome through phagocytosing membrane proteins or isolated fragments of organelles, which subsequently fuses with lysosome for degradation (Mizushima et al., 2010).

The 18 kDa translocator protein (TSPO) is a protein mainly located in the outer membrane of mitochondria in peripheral organs and brain, which plays an important role in regulating cognitive function (Yasin et al., 2017). A large number of *in vitro* studies have shown that the classic TSPO ligand PK11195 can reduce the activation of microglia, improve the cognitive function of rats and play a neuroprotective role (Ryu et al., 2005; Veiga et al., 2007; Ma et al., 2016). In this study, we used intraperitoneal LPS injection to establish a rat model of PND. We investigated the protective effect of PK11195 in the hippocampus of PND rats. This study provides an understanding on the mechanism of PK11195 in improving cognitive dysfunction.

## Materials and Methods

### Animals

Male, Sprague-Dawley, specific-pathogen-free rats (6–8 weeks old, weighing 200 ± 20 g) were provided by Pengyue Experimental Animal Breeding Co., Ltd. (Jinan, China). Rats were bred under standardized conditions and allowed to adapt to the environment for a week before experiments. All rats were treated in accordance with the Guidelines for the Care and Use of Laboratory Animals of the Ministry of Health of China and approved by the Animal Laboratory Center of Weifang Medical University.

### Experimental Groups and Drug Treatment

All rats were randomized into four groups (n = 20 in each group): control group (Con group), PK11195 group, LPS (PND model) group, PK11195 + LPS group. PND rat model was established by intraperitoneal injection of LPS (Product purity ≥ 99%; Solarbio, Beijing, China) 1 mg/kg for three consecutive days (Anaeigoudari et al., 2016). In the PK11195 group, rats were treated with PK11195 (Product purity ≥ 98%; Sigma, MO, United States) 3 mg/kg. In the PK11195 + LPS group, rats were pretreated with PK11195 30 min before LPS treatment. The animals of the control and LPS groups received 3 mg/kg of saline instead of PK11195. The animals of the control and PK11195 groups received 1 mg/kg of saline instead of LPS.

### Morris Water Maze (MWM) Test

After the rats adapted to the surrounding environment, the MWM test was performed using the instrument from Huaibei Zhenghua Biological Instrument Co., Ltd. (Anhui, China) to examine the spatial learning and memory capacities of the rats. In brief, rats were randomly placed into one of the three quadrants (except for the target quadrant) in a circular pool filled with water, and they are asked to find a transparent escape platform that is not visible in the center of the target quadrant. In an exploration test, each rat received a 5-day training with three trials per day using three different starting quadrants. Each rat was allowed to locate the platform submerged 1 cm below the water surface in 120 s. The swim velocity and the time to locate the platform (escape latency and the swim distance, defined by a cut-off time of 120 s) was recorded. On the sixth day, a probe trial was conducted with no escape platform and the number of times that the rat crossed the original platform site and the total time spent in the target quadrant were recorded by the tracking system.

### Western Blotting Analysis

The rat hippocampus was collected immediately after the animals completed the behavioral tests. Proteins of the tissues were extracted and the concentrations determined by BCA Protein Assay Kit (CWBIO, Beijing, China). Each sample contained with 40 μg of protein was separated by 10 or 12% SDS-PAGE and then transferred to nitrocellulose membranes (Millipore, MA). The following primary antibodies were incubated with the PVDF membrane at 4°C overnight. PBR (1:7,000 dilution, ab92291; Abcam), ATG7 (1:7,000 dilution, ab133528; Abcam), ATG5 (1:7,000 dilution, ab108327; Abcam), LC3B (1:2,000 dilution, ab192890; Abcam), p62 (1:7,000 dilution, ab109012; Abcam) and β-actin (1:7,000 dilution, aa128; Beyotime Biotechnology). After that, rabbit anti-goat immunoglobulin G (IgG) (H + L) horseradish peroxidase (HRP) (1:5,000 dilution, ZB2306; ZSGB-BIO), goat anti-rabbit immunoglobulin G (IgG) (H+L) HRP (1:7,000 dilution, GAR007; MultiSciences) or goat anti-mouse IgG (H+L) HRP (1:7,000 dilution, GAM007; MultiSciences) were incubated with the membrane for 2 h at room temperature. Bands were visualized with enhanced chemiluminescence (ECL) detection reagents (CWBIO, Beijing, China) using an ECL reagent. The relative band intensity was measured by Image-Pro Plus software.

### Quantitative Real-Time PCR (qRT-PCR)

Total RNA of the hippocampus samples was manually extracted using the TRIzol Reagent (ThermoFish Scientific, Shanghai, China). Then, the quantity of the RNA was determined using UV absorbance at 260 nm. Subsequently, reverse transcription was performed by using a ReverTtra Ace qRT-PCR Kit (Toyobo, Osaka, Japan) according to the manufacturer’s instructions. For qRT-PCR analysis, the resultant cDNA products were amplified using the 2×ChamQ SYBR qPCR Master Mix in triplicate. β-actin was used as an internal reference for amplification reaction. PCR amplification was performed with a program of 95°C for 1 min, followed by 40 cycles of 95°C for 15 s, and 60°C for 25 s. The primer sequences were listed in Table 1.

**TABLE 1:** Primer sequences for qRT-PCR.

Gene	Forward (5′ → 3′)	Reserve (5′ → 3′)
β-actin	CTA​AGG​CCA​ACC​GTG​AAA​AG	ACC​AGA​GGC​ATA​CAG​GGA​CA
PBR	CTA​CTT​TGT​GCG​TGG​TGA​GGG	AGA​CCC​AAG​GGA​ACC​ATA​GCC
ATG5	TGA​CCA​GTT​TTG​GAC​CAT​CA	AGG​GTA​TGC​AGC​TGT​CCA​TC
ATG7	ATG​CCA​GGA​CAC​CCT​GTG​AAC​TTC	ACA​TCA​TTG​CAG​AAG​TAG​CAG​CCA
LC3B	GAA​GAC​CTT​CAA​ACA​GCG​CC	CTT​GGT​CTT​GTC​CAG​GAC​GG
p62	ATGGACATGGGGAGCTICAA	GTG​CTC​TCT​GTA​TGC​TCC​CT

### Laser Confocal Microscopy

The rats were anesthetized and perfused with 4% paraformaldehyde, then the brain tissues were collected, fixed and sliced. Next, 10% normal donkey or goat blocking serum (Solarbio, Beijing, China) was used to block brain tissues sections for 30 min at 37°C. The sections (10 μm) were then incubated at 4°C overnight with the following primary antibodies: TSPO (1:100 dilution, pa5-19088; ThermoFish Scientific), Iba1 (1:200 dilution, gb12105; Servicebio), ATG7 (1:100 dilution, ab133528; Abcam), LC3B (1:100 dilution, ab192890; Abcam) and p62 (1:100 dilution, ab109012; Abcam). The secondary antibodies Cy3 conjugated Donkey Anti-Mouse IgG (H+L) (1:200 dilution, gb21401; Servicebio), FITC conjugated Donkey Anti-Goat IgG (H+L) (1:200 dilution, gb22404; Servicebio), Alexa Fluor 488-conjugated Goat Anti-Rabbit IgG (H+L) (1:400 dilution, gb25303; Servicebio) and Cy3 conjugated Goat Anti-mouse IgG (H+L) (1:300 dilution, gb21301; Servicebio) were used and incubated 1 h in the dark. The sections were incubated with Hoechst (1:600 dilution, g1011; Servicebio) for 3 min for nuclear staining. The sections were analyzed and photographed using a laser scanning confocal microscope (NIKON Eclipse Ti, Japan). Finally, Image J performs quantitative analysis.

### Transmission Electron Microscopy

In order to study the ultrastructure, coronal slices of rat brain tissue were prepared according to the stereotaxic coordinates of hippocampus and soaked immediately in 3% cold glutaraldehyde phosphate at 4°C for 6–8 h, then they were cut into 1 mm^3^ blocks, washed with 0.1 M PBS (pH 7.4), fixed in 1% osmium tetroxide (OsO4) for 1–2 h. The dehydration of blocks was carried out with upgraded alcohol and acetone series. Next, the blocks were embedded in the epoxy resin at 60°C for 48 h. Subsequently, ultrathin sections were cut and double stained with 4% uranium acetate and 0.5% lead citrate. Finally, the ultrastructural characteristics of hippocampal CA1 microglia were observed under the TEM (HT 7700-SS, HITACHI, Japan).

### Statistical Analysis

All data were expressed as a mean ± standard deviation (SD) and analyzed using SPSS 20.0. Data in the Morris water maze studies were analyzed using two-way analysis of variance (ANOVA) with repeated measures. Other data were analyzed via one-way ANOVA followed by Tukey’s post-hoc test. A *p* value of less than 0.05 served as the criterion for statistical significance.

## Results

### PK11195 Reduces LPS-Induced Learning and Memory Impairment

**FIGURE 1:**
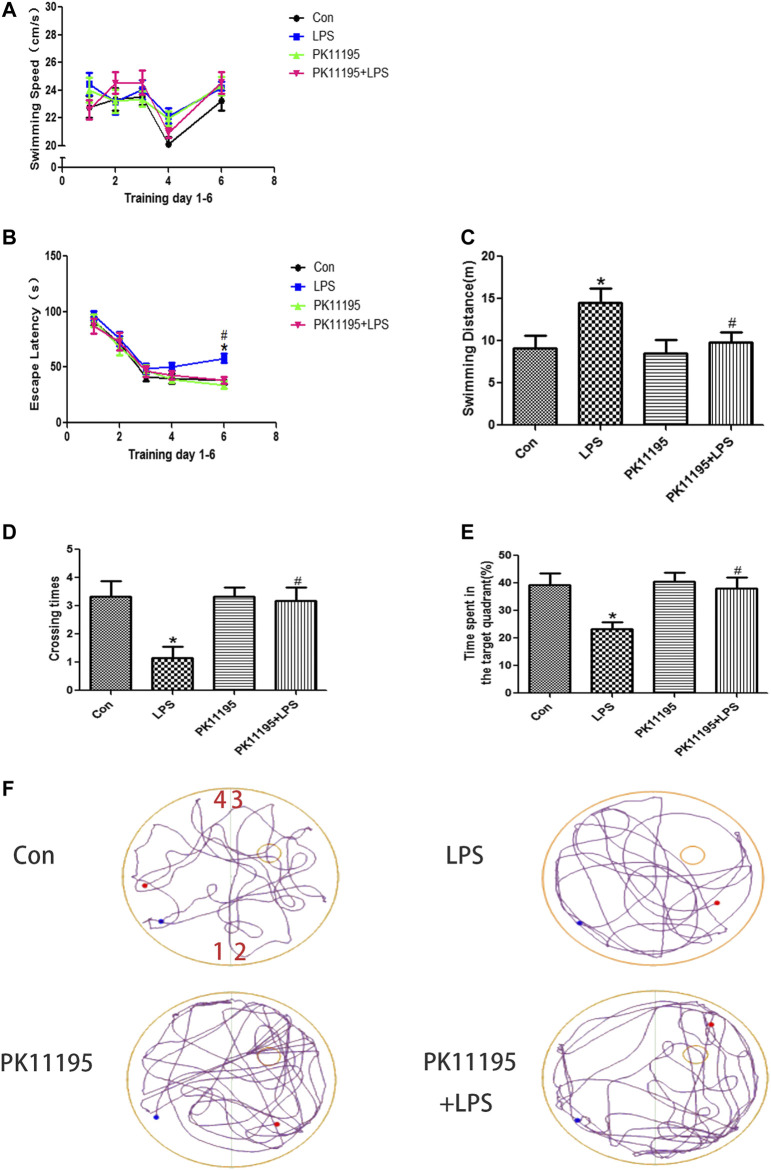
Spatial learning and memory ability of rats in Morris water maze test. **(A)** swimming speed; **(B)** escape latency; **(C)** swimming distance on the sixth day; **(D)** crossing times; **(E)** time spent in the target quadrant; **(F)** the representative trajectory diagrams (1, 2, 3, 4: four different quadrants; Blue dots: starting position; Red dots: ending position; Small yellow circles: the previous location of the platform.); Values are presented as the mean ± standard deviation. *p < 0.05 vs. Con group; ^#^ *p* < 0.05 vs. LPS group (n = 10 in each group). LPS induced the expression of TSPO in the hippocampus, PK11195 attenuate this response.

The results of MWM tests showed that, there was no significant difference in swimming speed among the four groups (*p* > 0.05) (Figure 1A). The rats in the LPS groups had higher escape latency and longer swimming distance than rats in the Con group on the sixth day (*p* < 0.05). The escape latency and swimming distance of the PK11195 + LPS group was significantly reduced compared with the LPS group on the sixth day (*p* < 0.05) (Figures 1B,C). The results of the probe trail showed that the rats in the LPS groups crossed the transparent platform fewer times and spent less time in the target quadrant searching for the missing platform than rats in the Con group on the sixth day (*p* < 0.05). The number of platform crossings and the total time spent in the target quadrant of the PK11195 + LPS group was significantly increased compared with the LPS group on the sixth day (*p* < 0.05) (Figures 1D,E). The representative trajectory diagrams are shown in Figure 1F.

### LPS Induced the Expression of TSPO in the Hippocampus, PK11195 Attenuate This Response

**FIGURE 2:**
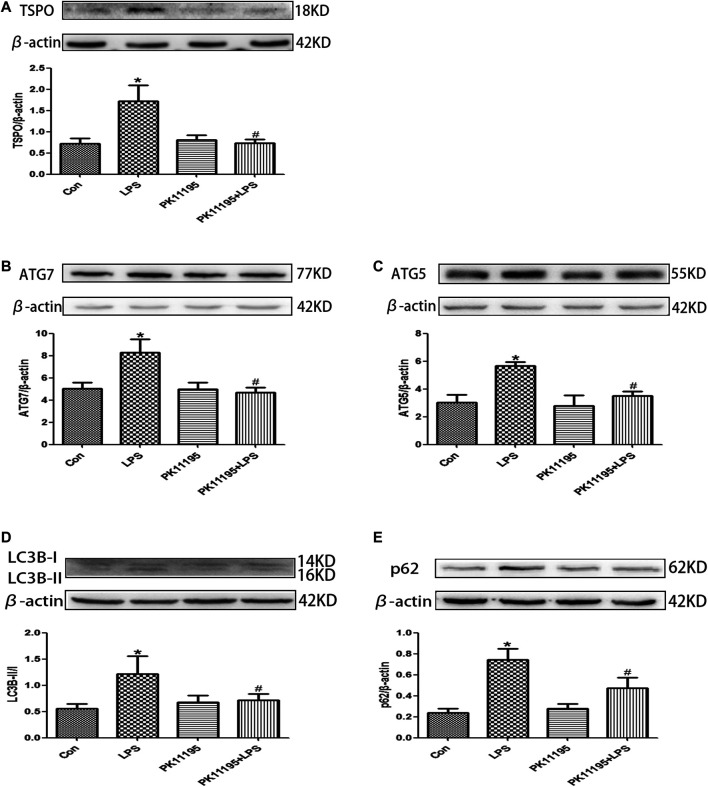
The expression of TSPO and autophagy-related proteins were quantifified by Western blotting in the hippocampus. **(A)** Bar graphs showing TSPO protein expressions in the hippocampus. Statistical analysis graph of each group indicating TSPO protein levels in the hippocampus; **(B)** Bar graphs showing ATG7 protein expressions in the hippocampus. Statistical analysis graph of each group indicating ATG7 protein levels in the hippocampus; **(C)** Bar graphs showing ATG5 protein expressions in the hippocampus. Statistical analysis graph of each group indicating ATG5 protein levels in the hippocampus; **(D)** Bar graphs showing LC3Bll/l protein expressions in the hippocampus. Statistical analysis graph of each group indicating LC3Bll/l protein levels in the hippocampus; **(E)** Bar graphs showing p62 protein expressions in the hippocampus. Statistical analysis graph of each group indicating p62 protein levels in the hippocampus. Values are presented as the mean ± standard deviation. *p < 0.05 vs. Con group; #*p* < 0.05 vs. LPS group (n = 5 in each group).

**FIGURE 3:**
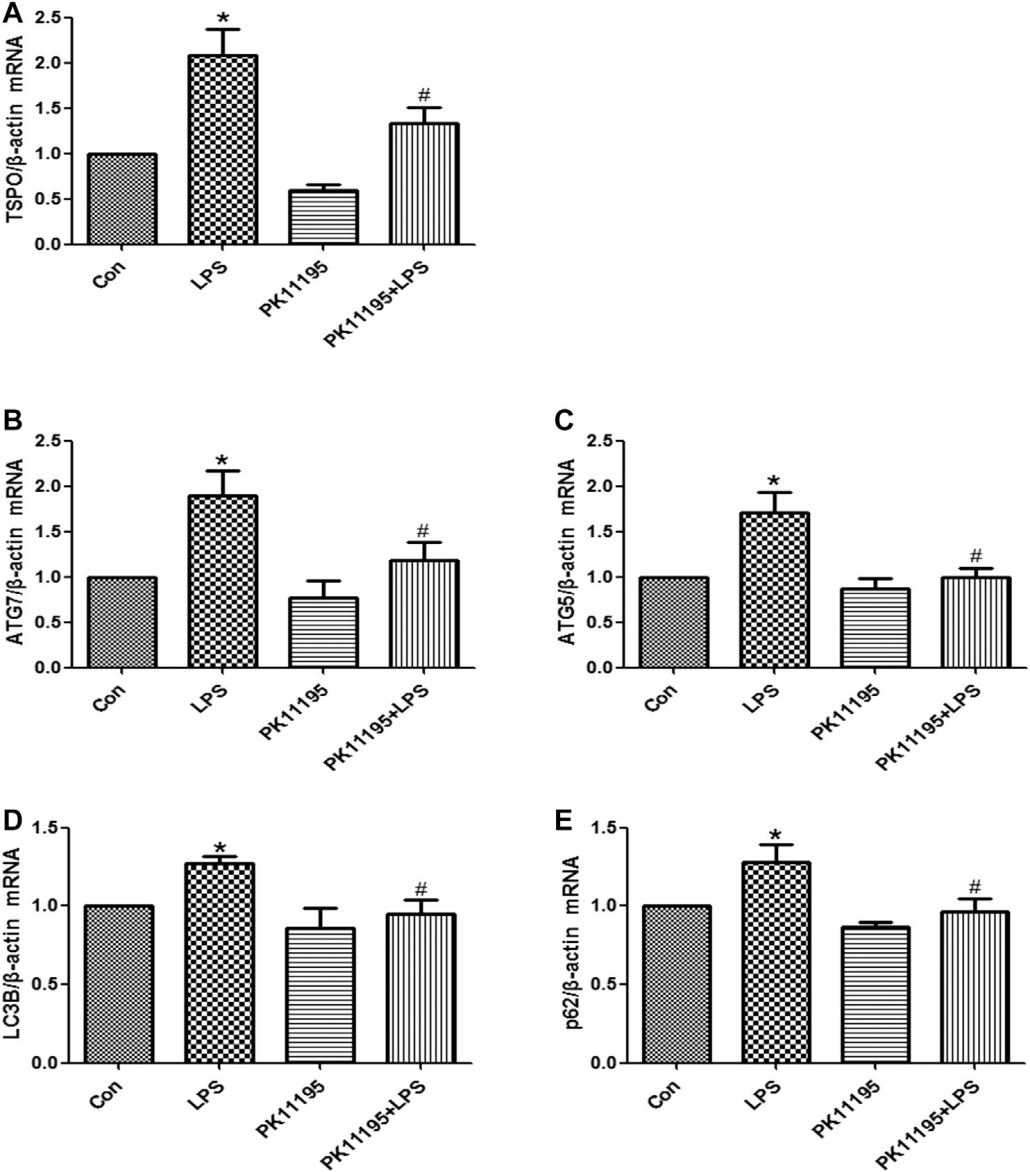
The expression of TSPO and autophagy‐related mRNA were quantifified by qRT‐PCR in the hippocampus. **(A)** Statistical analysis graph of each group indicating TSPO mRNA levels in the hippocampus; **(B)** Statistical analysis graph of each group indicating ATG7 mRNA levels in the hippocampus; **(C)** Statistical analysis graph of each group indicating ATG5 mRNA levels in the hippocampus; **(D)** Statistical analysis graph of each group indicating LC3B mRNA levels in the hippocampus; **(E)** Statistical analysis graph of each group indicating p62 mRNA levels in the hippocampus. Values are presented as the mean ± standard deviation. *p > 0.05 vs. Con group; #*p* < 0.05 vs. LPS group (n = 5 in each group).

**FIGURE 4:**
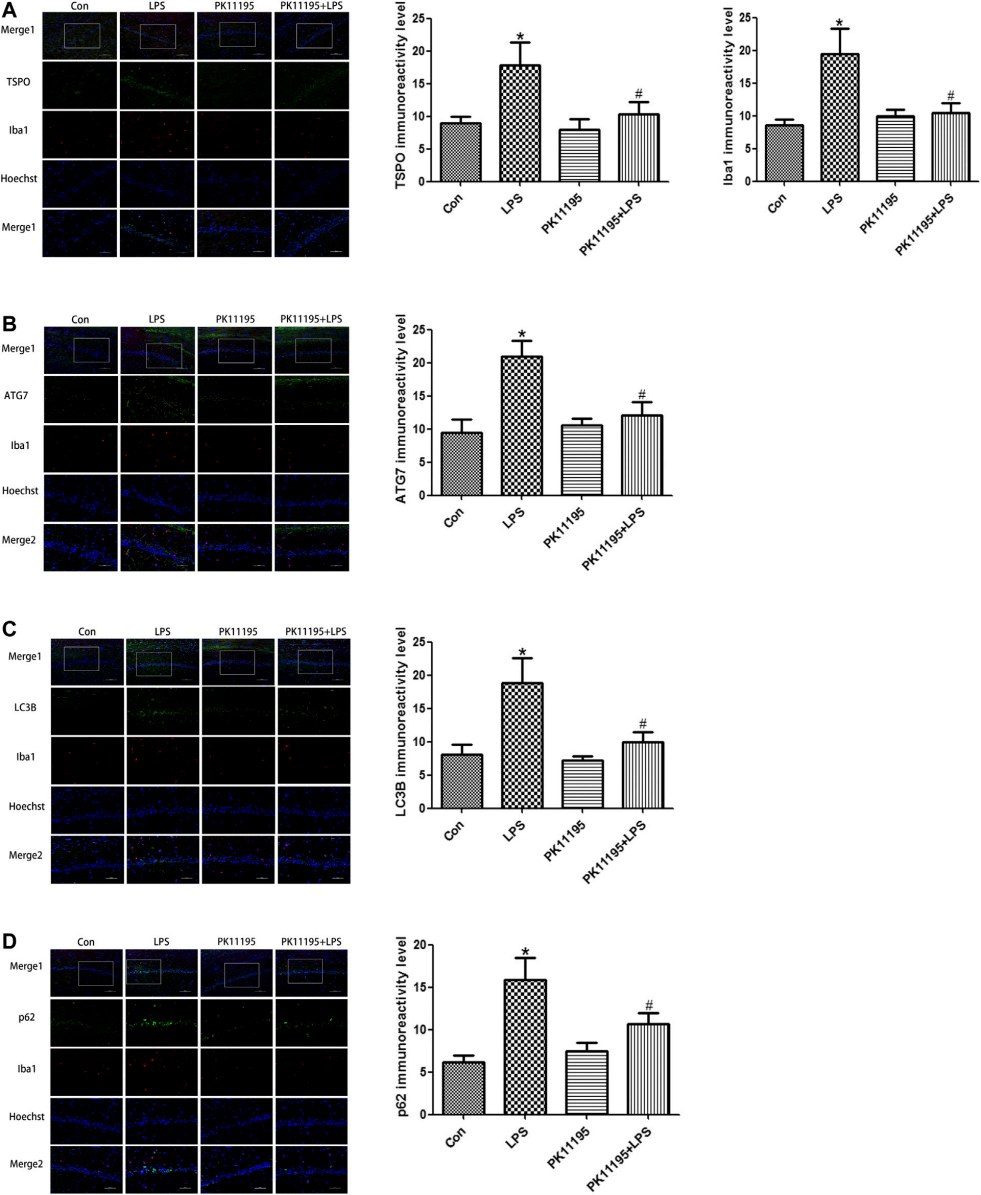
Laser confocal microscopy showing the expression of TSPO, Iba1 and autophagy-related proteins in the rat hippocampal CA1 microglia of each group (Merge1: Bar = 100 μm, ×200, the rest: Bar = 50 μm, ×400). **(A)** Statistical analysis graph of each group indicating TSPO and Iba1 protein levels in the rat hippocampal CA1 microglia; Green represents TSPO, red represents Iba1 and blue represents the nuclei. **(B)** Statistical analysis graph of each group indicating ATG7 protein levels in the rat hippocampal CA1 microglia; Green represents ATG7, red represents Iba1 and blue represents the nuclei. **(C)** Statistical analysis graph of each group indicating LC3B protein levels in the rat hippocampal CA1 microglia; Green represents LC3B, red represents Iba1 and blue represents the nuclei. **(D)** Statistical analysis graph of each group indicating p62 protein levels in the rat hippocampal CA1 microglia; Green represents p62, red represents Iba1 and blue represents the nuclei. Values are presented as the mean ± standard deviation. *p < 0.05 vs. Con group; #*p* < 0.05 vs. LPS group (n = 5 in each group).

The Western blot and qRT-PCR results show that the expression of TSPO protein and mRNA in the LPS group was significantly higher than that in the Con group (*p* < 0.05). The expression of the TSPO protein and mRNA in the PK11195 + LPS group was significantly lower than that in the LPS group (*p* < 0.05) (Figures 2A, 3A). Similarly, the results of laser confocal microscopy showed that the fluorescence intensity of TSPO and Iba1 was increased in the LPS group. The fluorescence intensity of TSPO and Iba1 in the PK11195 + LPS group was significantly lower than that of LPS group (Figure 4A). These results suggested that PK11195 reduce the activation of microglia in the hippocampus.

### PK11195 Alleviated Cognitive Impairment of Rats via Inhibiting Autophagy

**FIGURE 5:**
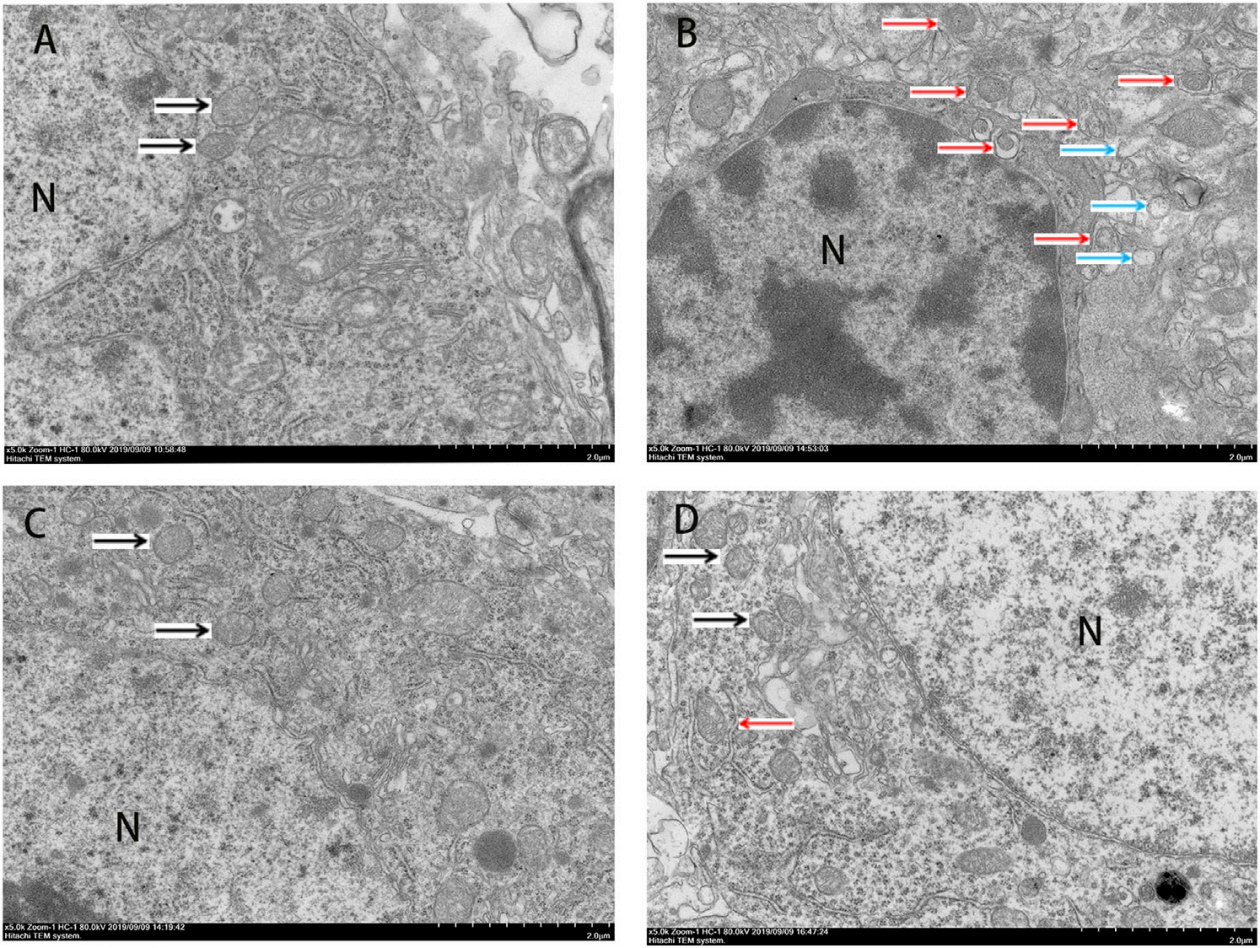
Transmission electron microscopy (TEM) was employed to detect the ultrastructure of rat hippocampal microglia (Bar = 2 μm). **(A)** Con; **(B)** LPS; **(C)** PK11195; **(D)** PK11195 + LPS; N, Nucleus. Black arrow: normal mitochondria, Red arrow: autophagosome, Blue arrow: phagocytosis.

The Western blot and qRT-PCR results showed that PK11195 reduced cognitive impairment of rats via inhibiting autophagy. The expression of ATG7, ATG5, LC3B and p62 protein and mRNA increased in the LPS group compared with the Con group (*p* < 0.05). The expression of ATG7, ATG5, LC3B and p62 protein and mRNA in the PK11195 + LPS group was reduced compared with the LPS group (*p* < 0.05) (Figures 2B–E, 3B–E). Similarly, the results of laser confocal microscopy showed that the ATG7, LC3B and p62 protein intensity was significantly enhanced in the LPS group compared with Con group. The ATG7, LC3B and p62 protein intensity was significantly lower in the PK11195 + LPS group than the LPS group (*p* < 0.05) (Figures 4B–D). To further confirm that PK11195 reduced cellular autophagy, the ultrastructure of rat hippocampal microglia was imaged by transmission electron microscopy. Transmission electron microscopy results showed that compared with healthy mitochondria (black arrows) in the Con group (Figure 5A), mitochondria in the cytoplasm of microglia in the LPS group were severely damaged, and a large number of autophagosomes (red solid arrows) and phagocytic vesicles (blue solid arrows) are visible. The chromatin in the cell nucleus gathers into clusters, forming crescent-shaped and irregular high-electron-density clumps, which is distributed around the nuclear membrane (Figure 5B); In the PK11195 + LPS group, the structure of mitochondria in the cytoplasm of microglia is generally normal, and the number of autophagosomes and phagocytic vesicles decreased significantly; the chromatin distribution is relatively uniform (Figure 5D).

## Discussion

This study explored the potential mechanism that PK11195 improves cognitive function of PND animals. Our results showed that PK11195 may play a neuroprotective role to improve the cognitive function of rats by inhibiting microglial activation and mitochondrial autophagy.

PND is associated with surgical complications, prolonged hospitalization, impaired quality of life, and increased risk of disability and mortality (Steinmetz et al., 2009). PND is the main cause of post-operative disability and premature death (Wyss-Coray, 2016). Currently, effective clinical intervention to prevent this disorder is limited. In this study, it was found that continuous intraperitoneal injection of 1 mg/kg LPS caused cognitive dysfunction in rats which is in consistent with previous studies (Anaeigoudari et al., 2016), In addition, we used PK11195 at 3 mg/kg 30 min before LPS injection to establish a PK11195 + LPS rat model according to Ma’s protocol (Ma et al., 2016). Morris water maze tests showed that rats in the LPS group need to spend more time, longer distances to find the platform and fewer target quadrant platform crossing times and swimming times than the Con group. The rats in the PK11195 + LPS group spend less time, shorter distances to find the platform and more target quadrant platform crossing times and swimming times, and they are also better at remembering the position of the platform compared with the LPS group. These results indicate that LPS exposure cause learning and memory dysfunction in rats, and PK11195 pretreatment can improve the cognitive impairment caused by LPS exposure.

Previous studies have found that the expression of TSPO is significantly increased when the nervous system is damaged, especially in Alzheimer’s disease, Parkinson’s disease, Huntington’s disease and Amyotrophic Lateral Sclerosis (Turner et al., 2004; Ghadery et al., 2017; Simmons et al., 2018; Gui et al., 2020). These studies suggest that TSPO plays an important role in the pathophysiology of neurodegeneration. Overexpression of TSPO in reactive microglia is considered as a hallmark of neurodegeneration (Venneti et al., 2006; Hannestad et al., 2012). TSPO is considered to be both a diagnostic biomarker and a therapeutic target for neurodegenerative diseases (Rupprecht et al., 2010). In various experimental injury and disease models, TSPO ligands have been shown to inhibit the activation of microglia, increase the survival rate of neurons and improve the regeneration process (Ryu et al., 2005; Veiga et al., 2005). It was known that TSPO is mainly expressed in microglia in the central nervous system. The expression of TSPO is low in resting microglia, but when microglia is activated, its expression increases significantly (Papadopoulos et al., 2006; Karlstetter et al., 2014). Some studies have shown that TSPO specific ligands protect animal models from brain damage and have potential therapeutic value for repairing brain damage (Palzur et al., 2016; Li et al., 2017). The Western blot and qRT-PCR results show that compared with Con group, the expression of TSPO protein and mRNA in the LPS group increased significantly, PK11195 pretreatment can reduce the expression of TSPO protein and mRNA. Similarly, the results of laser confocal microscopy showed that the fluorescence intensity of TSPO and Iba1 increased in the LPS group. The fluorescence intensity of TSPO and Iba1 in the PK11195 + LPS group was significantly lower than LPS group. The above results indicate that PK11195 can inhibit the activation of microglia and the expression of TSPO protein and mRNA induced by LPS.

Mitochondrial autophagy plays an important role in maintaining the integrity of mitochondrial function, the stability of intracellular environment and cell survival (Lemasters, 2005). Autophagy is critical for the development and maintenance of the nervous system (Ye et al., 2016; Wu et al., 2017). However, with the enhancement of adverse external stimuli, when autophagy is insufficient to remove the damaged mitochondria, the nerve cells will undergo apoptosis. During the autophagosome elongation process, the soluble autophagosome light chain 3 (LC3I) is the autophagosome related form (LC3II) (Xiong et al., 2017). After fusion with lysosome, LC3II was degraded together with other components of autophagosome. Therefore, the transformation of LC3 or the production of LC3II have been used as biomarkers of autophagy activity (Klionsky et al., 2016). ATG7 is part of the conjugation system involved in the redistribution of LC3-II to the phagophore, and ATG5 covalently binds to ATG12 to constitute the ATG12–ATG5-ATG16 conjugation system, which is essential for autophagosome elongation (Montiel et al., 2020). During autophagy, p62 binds to ubiquitinated protein and forms a complex with LC3II protein located on the membrane of autophagosome, which is degraded in autophagic lysozyme. p62 is one of the biological marker proteins reflecting autophagy activity, and its content indirectly reflects the clearance level of autophagosome (Jang, 2020). Therefore, when autophagy occurs, the p62 protein is continuously degraded in the cytoplasm. However, when there is excessive autophagy or insufficient clearance of autophagosomes, p62 protein will continue to accumulate in the cytoplasm. This study found that the expression of ATG7, ATG5, LC3B protein and mRNA increased after LPS treatment, and PK11195 pretreatment reduced the expression of these autophagy-related proteins and mRNA. Similarly, the results of laser confocal microscopy showed that compared with the control group, the ATG7, LC3B and p62 protein intensity was significantly enhanced in the LPS group. The ATG7, LC3B and p62 protein intensity was significantly lower in the PK11195 + LPS group than in the LPS group. In TEM, we observed numerous damaged mitochondrias in the rat hippocampal microglia in the LPS group. The accumulated autophagosomes cannot be eliminated by autophagolysosome timely, aggravating cell burden and interrupting cell homeostasis, eventually triggering cell death. The PK11195 pretreatment improved the situation, including that the structure of mitochondria in the cytoplasm is generally normal, and the number of autophagosomes and phagocytic vesicles decreased significantly; the chromatin distribution is relatively uniform. These results indicate that TSPO ligand PK11195 pretreatment significantly reduces the autophagy level of microglia and improves cognitive function in rats. Interestingly, LPS exposure can induce a significant increase in the autophagy substrate protein p62, which indicates a decrease in autophagosome clearance. This result is in line with Cui’s study (Cui et al., 2018), in which the expression of autophagosome-forming protein ATG7 and autophagy substrate p62 is up-regulated, suggesting that the increased autophagosomes induced by LPS is not only due to the enhancement of formation but also the decline in clearance.

In summary, this study showed that microglial autophagy autophage increased in the LPS-induced PND rat model. TSPO ligand PK11195 might improve the cognitive function of rats by inhibiting the activation of microglia and autophagy of mitochondria. This study provides new ideas and directions for clinical treatment of some neurological diseases and helps finding new strategies for prevention and treatment of neurodegenerative diseases.

## Data Availability

The raw data supporting the conclusions of this article will be made available by the authors, without undue reservation, to any qualified researcher.
